# The threshold effects of meteorological factors on Hand, foot, and mouth disease (HFMD) in China, 2011

**DOI:** 10.1038/srep36351

**Published:** 2016-11-16

**Authors:** Zhicheng Du, Wangjian Zhang, Dingmei Zhang, Shicheng Yu, Yuantao Hao

**Affiliations:** 1 Department of Medical Statistics and Epidemiology & Health Information Research Center & Guangdong Key Laboratory of Medicine, School of Public Health, Sun Yat-sen University, Guangzhou 510080, Guangdong Province, China; 2 Chinese Center for Disease Control and Prevention, Beijing 102206, China

## Abstract

We explored the threshold effects of meteorological factors on hand, foot and mouth disease (HFMD) in mainland China to improve the prevention and early warning. Using HFMD surveillance and meteorological data in 2011, we identified the threshold effects of predictors on the monthly incidence of HFMD and predicted the high risk months, with classification and regression tree models (CART). The results of the classification tree showed that there was an 82.35% chance for a high risk of HFMD when the temperature was greater than 24.03 °C and the relative humidity was less than 60.9% during non-autumn seasons. According to the heatmap of high risk prediction, the HFMD incidence in most provinces was beyond the normal level during May to August. The results of regression tree showed that when the temperature was greater than 24.85 °C and the relative humidity was between 80.59% and 82.55%, the relative risk (RR) of HFMD was 3.49 relative to monthly average incidence. This study provided quantitative evidence for the threshold effects of meteorological factors on HFMD in China. The conditions of a temperature greater than 24.85 °C and a relative humidity between 80.59% and 82.55% would lead to a higher risk of HFMD.

Hand, foot and mouth disease (HFMD) is a childhood infectious gastrointestinal disease that is commonly caused by enterovirus 71 (EV71) and coxsackie virusA16 (CV-A16)^1^,^2^. It has resulted in major outbreaks across the world in past three decades and become a major public health issue in China, affecting over two million persons annually^3^,^4^,^5^. The incidence and mortality of HFMD have been leading the type C infectious diseases since its first outbreak in China in 2008. The serious harm and the severe epidemic situation of HFMD have raised extensive attentions to its risk factors researching and control efforts.

The impacts of meteorological factors on HFMD have been studied extensively^1^,^2^,^6^,^7^,^8^. Meteorological predictors such as temperature, relative humidity and atmospheric pressure are most commonly reported by environmental agents, which may have significant effects on HFMD epidemics. Part of the studies speculated that there may exist a threshold for the effects^1^,^7^,^8^,^9^. For instance, Huang *et al*. found that the risk of HFMD increased sharply before about 25 °C and became relatively flat afterwards^1^. Zhang *et al*. found that two peaks exist on the humidex-incidence relationship, with one peak occurred at a humidex between 15 and 20, and the other was between 30 and 35^8^. Lin *et al*. found that an U-shape relationship was observed, with both high and low temperatures having an acute effect on HFMD risk^9^.

However, statistical models used in these researches were always difficult to cope with the collinearity problems and high-level interactions between meteorological factors^10^. And the same problems existed among meteorological factors were also discussed in the field of infectious diseases^11^. In addition, previous studies could not give the exact thresholds of the meteorological factors, which may be used for the public information and promoting the implementation research.

In order to make up for the inadequacy of existing research, this paper used a nonparametric modeling method, the classification and regression tree model (CART), to analyze HFMD surveillance data and meteorological data during 2011 in China. We tried to identify the threshold effects of predictors on HFMD after controlling the bias of collinearity problems and high-level interactions, and predict the high risk period of HFMD in China.

## Results

### Descriptive analysis

**Figure 1:**
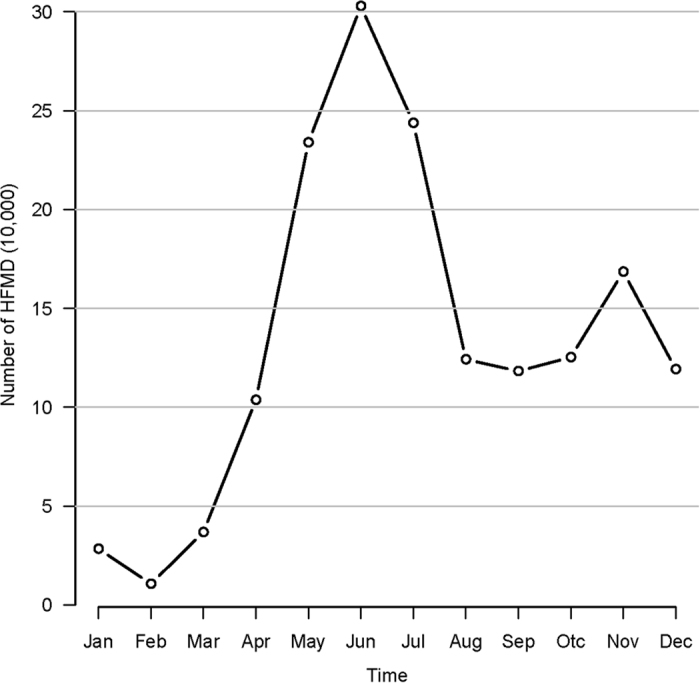
The HFMD counts between January 2011 and December 2011 in China.

Table 1 shows the descriptive statistics for each variable. The monthly mean incidence of HFMD, atmospheric pressure, relative humidity and temperature were 9.99(1/100,000), 95.37 (kPa), 63.07 (%) and 18.99 (°C), respectively. Figure 1 depicts the variation over time in HFMD between January and December in 2011. The curve indicates a seasonal pattern. About 95% of HFMD cases occurred in children under 5 years old in 2011.

### Association analysis

**Figure 2:**
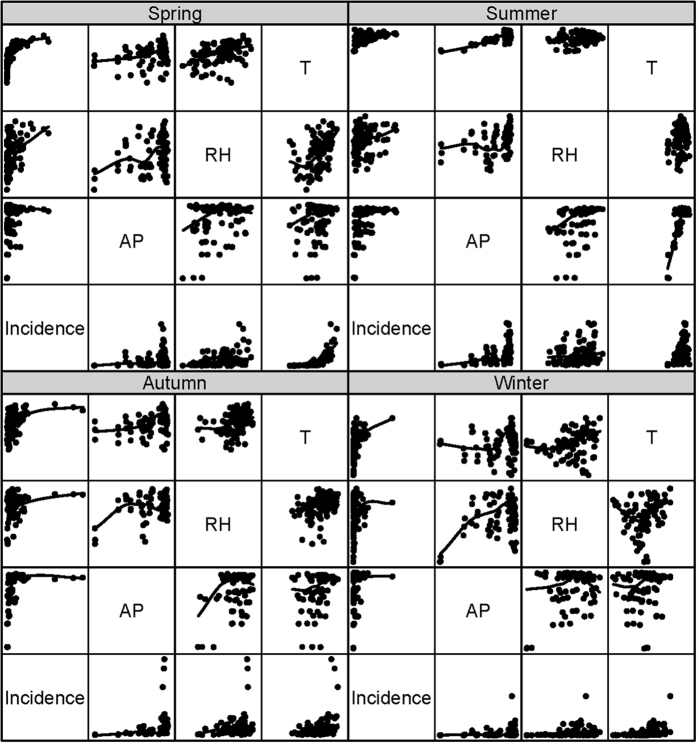
Scatter−plot matrices (with LOESS smooths) between HFMD incidences and explanatory variables.

Table 2 shows the bivariate linear relationships between the HFMD incidence and atmospheric pressure, relative humidity and temperature. Atmospheric pressure (*r* = 0.259, *P* < 0.001), relative humidity (*r* = 0.308, *P* < 0.001) and temperature (*r* = 0.488, *P* < 0.001) were significantly associated with HFMD incidence. The Scatter plot matrices with spline regression line depict the relationship between all the variables by season in Fig. 2.

There was a significant positive spatial autocorrelation of HFMD incidence with a Moran’s *I* statistic of 0.324 (*P* < 0.001).

### Spatial empirical Bayes rates smoothing

**Figure 3:**
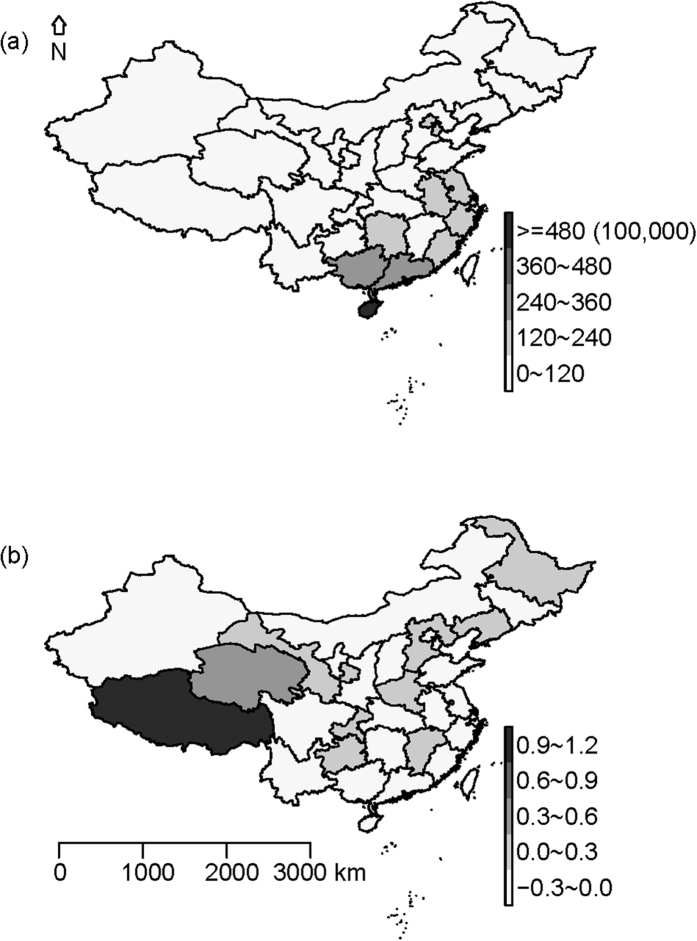
Choropleth map of notified incidence rates of HFMD (**a**) and the rates changed after spatial empirical Bayesian smoothed (**b**) (Maps were created with R version 3.3.0 (R Core Team, Vienna, Austria) package maptools version 0.8-39).

Figure 3(a) depicts the geographic distribution of the notified HFMD incidences in China during 2011. The figure confirms that the risk for HFMD varied with geographical location. The spatial empirical Bayes smoothing analysis (Fig. 3(b)) showed that the HFMD incidence changed a little (−0.3/100,000 to 1.2/100,000). And the infection activity was primarily concentrated in the southeast.

The Bayesian posterior estimates of the monthly HFMD incidence for each province would be used in the CART models.

### CART 1 model

**Figure 4:**
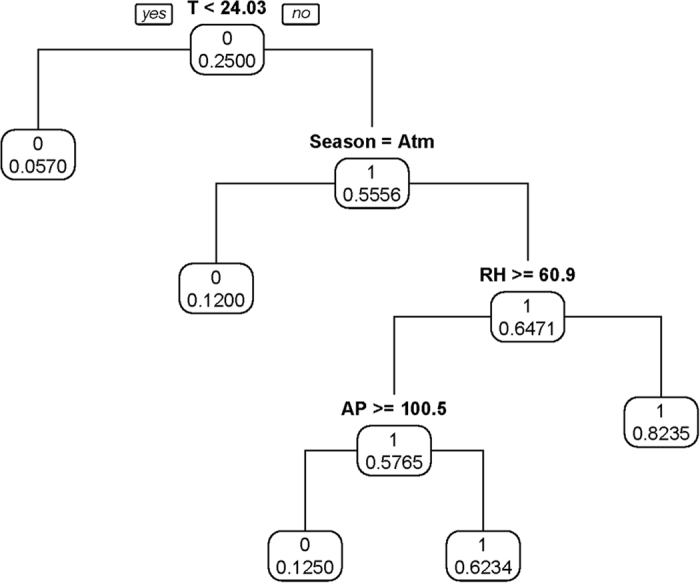
Results of CART model of probability of high risk.

Figure 4 representing the first CART model indicated the high risk period of HFMD, defined as exceeding the third quartile of the incidence. The analysis indicates that there was an 82.35% chance for a high-risk of HFMD when the temperature was greater than 24.03 °C and the relative humidity was less than 60.9% during non-autumn seasons.

**Figure 5:**
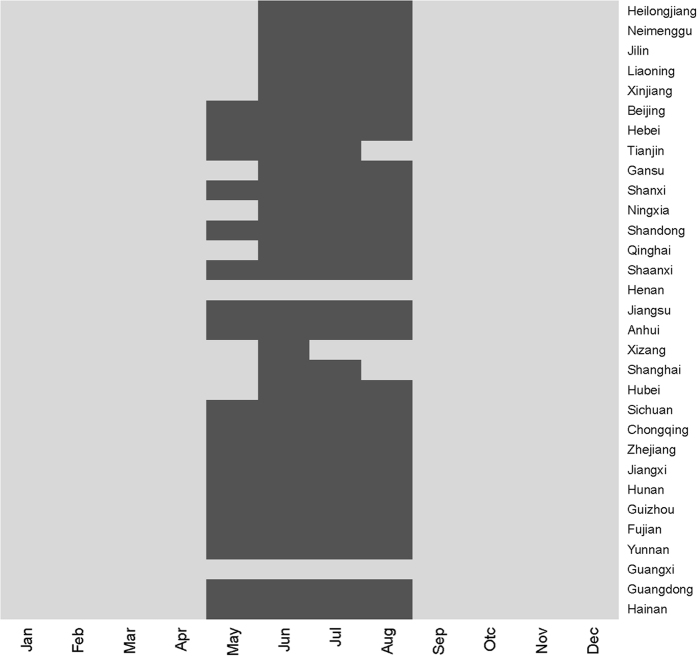
Heatmap of the high risk prediction based on CART1 model (black: high risk).

Figure 5 shows a high risk map of predicted monthly HFMD incidence rates in China based on the CART 1 model, which took into account local variation (i.e. atmospheric pressure, relative humidity, temperature and season) from the model prediction. Thirty one provinces were sorted in descending latitude from up to down. The heatmap shows that the detected high risk timing was concentrate from May to August. The time of south provinces was earlier than the north. The validation analysis indicates that the classification accuracy rate was 86.02%.

### CART 2 model

**Figure 6:**
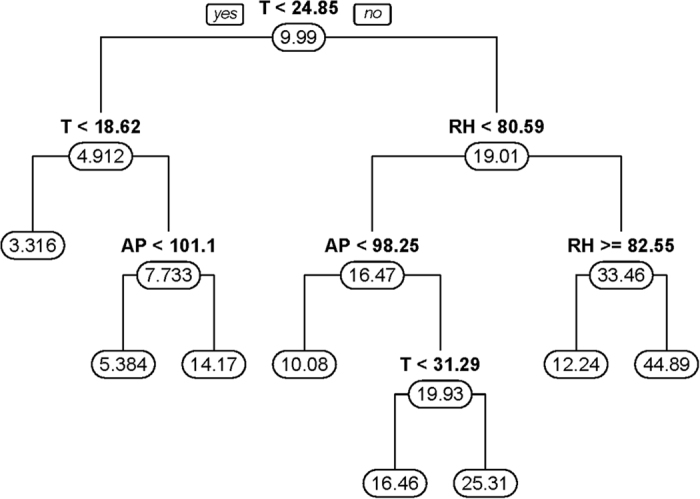
Results of CART model of expected incidence of HFMD.

Figure 6 represented the second CART model for the smoothing incidence of HFMD in a month, by province. The results indicated that when the temperature was greater than 24.85 °C and the relative humidity was between 80.59% and 82.55%, the relative risk (RR) of HFMD was 3.49 relative to monthly average incidence (mean incidence: 9.989/100,000) during the epidemic period. The result of the leave-one-out cross-validation (LOOCV) indicated that the prediction error of the model was 10.80%.

These results of validation analysis including misclassification analysis and LOOCV reveal that both the two CART models had reasonable accuracy, and its utility in research needs to be further explored.

## Discussion

The current study has quantified the threshold effects of meteorological factors on HFMD, using China as the study area in 2011. Because of the absence of HFMD-targeted vaccination or specific treatments, quantification of the adverse effects of environmental agents is essential for the early warning and response system on HFMD^7^,^12^.

Most parts of the world have reported the epidemic of HFMD. As the high prevalence area, China should be worthy of attention to the control work. Our results showed that HFMD in China had a significant seasonal periodicity, including a major peak in summer followed by a smaller peak in autumn. As suggested by this figure, the correlation between incidences and weather factors varied across the seasons. High temperature and high humidity were indicated as the risk factors, being in agreement with reports from other research^13^. And the season factor was used to handle the temporal correlation in our CART model.

The related researches on HFMD found that meteorological predictors were the potential risk factors^1^,^2^,^13^. What’s more, we have also analyzed the threshold effects. We found that conditions of a temperature greater than 24.03 °C and a relative humidity under 60.9% (CART 1) or of a temperature greater than 24.85 °C and a relative humidity between 80.59% and 82.55% (CART 2) would lead to a higher risk. These two factors accounted for most importance for the increasing incidence of HFMD. Seasons and the atmospheric pressure could also affect the epidemic of HFMD. The threshold effect can be a continuation and development for the researches on HFMD. The meteorological is one of the major factors in the development of HFMD. They not only affect the individual immunity, but also affect the reproduction and transmission of the virus and media^14^,^15^.

This study stood out from previous studies by giving the exact thresholds of predictors to make the statistical results more straightforward to understand and reuse. With the two-stage CART models using the same predictors, two sets of thresholds were obtained for the different purposes. However, they were basically agreed with another. It meant that the thresholds we found were reliable, although the CART model allowed the high level interactions among predictors.

The acceptable results of validation analysis for the thresholds indicated another strength of our study. With the CART 1 model, high-risk months of each province in 2011 were predicted. The validation analysis showed that the classification accuracy rate was high (86.02%). The LOOCV result of the regression tree also indicated a high precision with an error of 10.80%. Different from other relevant studies, we provide the threshold of the predictors. We will conduct vigorous information and take emergency response plan before the meteorological factors reach the thresholds.

Limitations of this study should also be acknowledged. Firstly, we handled the temporal and spatial correlations in a simple way which was independent from the model. Much more difficult job should be done to improve algorithm of the model^16^. Secondly, the socio-economic factors should be taken into account to improve the prediction accuracy. Thirdly, underreporting of HFMD cases is inevitable because of the characteristics of being self-limiting, even though the data quality of the surveillance system in China was assured^13^. Finally, without information of changes of confounders including demographics and adaptation, results of this study should be extrapolated with caution. However, the methods used in our studies could serve as a good reference.

## Conclusion

This study provided quantitative evidence for the threshold effects of meteorological factors on HFMD in China. The incidence of HFMD in China was associated with temperature and relative humidity. The overlap threshold between these two-stage models was that conditions of a temperature greater than 24.85 °C and a relative humidity between 80.59% and 82.55% would lead to a higher risk of HFMD. These information could be helpful in predicting the scale of outbreaks, guiding health resource allocation and building public health preparedness and intervention strategies. Similar studies with threshold effects are needed to better understand the impacts of meteorological variables on HFMD.

## Materials and Methods

### Ethics statement

This study was based on official hand, foot and mouth disease (HFMD) surveillance data in China. Analyses were conducted at aggregate level and no confidential information was involved. The research study protocol was approved by the Institutional Review Board of the School of Public Health, Sun Yat-sen University. All methods were performed in accordance with the relevant ethical guidelines and regulations.

### Study area

China mainland consists of thirty one provinces which are available in many datasets, with populations per province ranging in size from 3.03 to 105.05 million. The meteorological characteristics vary regionally and seasonally among the country, with six zones including the tropical, subtropical, warm-temperate, mid-temperate, cold-temperate and the Tibet zone. In general, it performs complex landforms and climates in the study area.

### Data collection

We obtained the computerized data set on the reported HFMD cases by provinces in China for the period of 1^st^ January–31^st^ December 2011 from the National Center for Public Health Surveillance and Information Services, China Center for Disease Control and Prevention (China CDC) (http://cdc.cma.gov.cn/). Meteorological data were obtained for the same period from the China Meteorological Data Sharing Service System (http://cdc.nmic.cn/home.do) which were comprised of monthly mean atmospheric pressure (kPa), monthly mean relative humidity (%), monthly mean daily maximum temperature (°C).

### Spatial autocorrelation analysis

Moran’s *I* spatial autocorrelation statistic was calculated to determine whether spatial clustering was a feature of HFMD. Moran’s *I* is defined by:


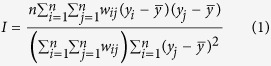


*y*_*i*_ and *y*_*j*_ denote the observed value at location *i* and *j*, 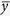 is the average of the *y* values over the *n* locations, and *w*_*ij*_ is the spatial weight measure^17^. Moran’s *I* can be interpreted as follows: a value close to 0 indicates randomness, while a positive (negative) value indicates positive (negative) autocorrelation.

The Moran’s *I* spatial autocorrelation statistic was calculated using the R package *sp* version 1.2-2^18^ and *spdep* version 0.5-92^19^.

### Spatial empirical Bayes rates smoothing

Bayesian conditional autoregressive (CAR) was used to obtain the more stable rates, improving the credibility of disease mapping^20^. Spatial empirical Bayes method defines a neighborhood for each study area (namely spatial weight matrix), and builds the risk estimation model by taking the scope of neighborhood, neighborhood population and mean and variance of neighborhood incidence into account. The Bayesian CAR could be implemented as a generalized linear mixed model:


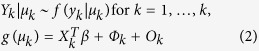


The study region *S* was partitioned into *k* non-overlapping areal units *S* = {*S*_1_, …, *S*_*k*_}, which were linked to a corresponding set of responses *Y* = {*Y*_1_, …, *Y*_*k*_}^*T*^, and a vector of known offsets *O* = {*O*_1_, …, *O*_*k*_}^*T*^. A set of random effects *Φ* = {*Φ*_1_, …, *Φ*_k_} were included to model any spatial autocorrelation in the data. The responses *Y*_*k*_ come from an Poisson family of distributions *f*(*y*_*k*_|*μ*_*k*_, *v*^2^). The expected value of *Y*_*k*_ is denoted by E(*Y*_*k*_) = *μ*_*k*_. The expected values of the responses are related to the linear predictor via an invertible link function *g*(·), and in our study natural log function was used and no predictor 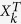 in the smoothing procedure.

For the global smoothing CAR priors, the model proposed by Leroux *et al*. (1999) was given by^21^:


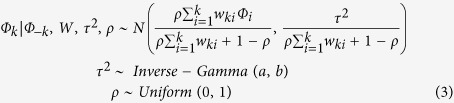


where *W* is a non-negative symmetric *k* × *k* neighborhood matrix. *ρ* is a spatial autocorrelation parameter, with *ρ* = 0 corresponding to independence, while *ρ* = 1 corresponds to strong spatial autocorrelation. A uniform prior on the unit intervalis specied for *ρ* while the usual inverse gamma prior is adopted for *τ*^2^. The default prior speciation for *τ*^2^ has *a* = *b* = 0.001.

The spatial empirical Bayes rates smoothing analysis was conducted using the R package *CARB ayes* version 4.4^22^.

### Spatiotemporal CART models

We considered a suite of two spatiotemporal CART models: (1) fitting a classification tree to incidences categorized as binary: high risk or non-high risk; (2) fitting a regression tree to the incidences.

### CART1: fitting a tree to high risk of HFMD

We defined a high risk if the incidence in any month exceeded the third quartile of the incidence per province in China during the 12 months of 2011. The spatiotemporal CART model was thus described as: monthly HFMD high risk/non-high risk ~ atmospheric pressure + relative humidity + temperature + season. The results are reported as the probability of occurrence of a high risk HMFD per month. A map of predicted probabilities was developed using the outputs of the CART model.

### CART 2: fitting a tree to the incidences

Like a classification tree, a regression tree is also built by binary recursive partitioning, while the response variable is continuous^23^. The splitting rules used in the algorithm are based on minimizing the sum of the squared deviations from the mean in the two separate subgroups. The squared residuals minimization algorithm is identical to a Gini splitting rule used in the classification^23^. Monthly HFMD incidence (1/100,000) by province were used as a continuous response variable in this model. The second CART model was thus described as: monthly HFMD incidence ~ atmospheric pressure + relative humidity + temperature + season. The results are reported as the RR of incidence, compared to average incidence rate of HFMD. RR = (expected incidence − mean of incidence)/mean of incidence.

Both the two CART analysis consisted of three basic steps. Firstly, a preliminary tree was grown by recursive data partitioning. Secondly, nested trees were formed by reducing the number of nodes in the tree (pruning) according to the complexity parameter. Thirdly, 10-fold cross-validation was used to address over-fitting and to identify the optimal tree with respect to its predictive ability. To control for the impact of seasonality, we decomposed the HFMD incidence into four seasonal categories^11^ (coded as Spring: March - April - May; Summer: June - July - August; Autumn: September - October - November; Winter: December - January - February). Finally, we validated the model with the leave-one-out cross-validation and misclassification rate for the classification tree. For each CART model, the predicted probabilities or relative risks can be presented as a map. A map of predicting high risk was developed using the outputs of the CART 1 model.

The statistical analysis and visualization were conducted using the R package *rpart* version 4.1-10 and *rpart.plot* version 1.5.3.

## Additional Information

**How to cite this article**: Du, Z. *et al*. The threshold effects of meteorological factors on Hand, foot, and mouth disease (HFMD) in China, 2011. *Sci. Rep.* **6**, 36351; doi: 10.1038/srep36351 (2016).

**Publisher’s note:** Springer Nature remains neutral with regard to jurisdictional claims in published maps and institutional affiliations.

## Figures and Tables

**Table 1:** Descriptive statistics of monthly HFMD and meteorological variables by province in China.

	Mean ± SD*	Median (IQR*)	Range
Incidence (1/100,000)	9.99 ± 12.88	6.16 (1.69–12.8)	(0.02–104.01)
atmospheric pressure (kPa)	95.37 ± 8.69	99.71 (91.77–100.99)	(64.66–103.24)
relative humidity (%)	63.07 ± 15.33	65.84 (53.98–74.3)	(15–90.03)
temperature (°C)	18.99 ± 10.84	21 (11.61–28)	(−16.17–36.77)

^*^SD: Standard Deviation; IQR: Inter-Quartile Range.

**Table 2:** Matrix of correlation coefficients between HFMD and meteorological factors.

	Incidence	Atmospheric pressure	Relative humidity
atmospheric pressure	0.259**		
relative humidity	0.308**	0.317**	
temperature	0.488**	0.057	0.222**

*P < 0.05, **P < 0.01.
